# Mitral Annular Systolic Velocities Predict Left Ventricular Wall Motion Abnormality During Dobutamine Stress Echocardiography

**DOI:** 10.4021/cr14w

**Published:** 2011-01-20

**Authors:** Dawod Sharif, Amal Sharif-Rasslan, Camilia Shahla

**Affiliations:** a Department of Cardiology, Bnai Zion Medical Center, Haifa, Israel; b Technion - Israel Institute of Technology, Haifa, Israel

**Keywords:** Stress echocardiogrpahy, Dobutamine, Tissue Doppler imaging, Ischemia, Coronary artery disease

## Abstract

**Background:**

Longitudinal systolic left ventricular contraction is complementary to the radial performance and can be assessed using tissue Doppler imaging (TDI). This study was performed to evaluate the contribution of mitral annular systolic velocities using TDI after dobutamine stress echocardiography (DSE).

**Methods and Results:**

Fifty subjects with suspected coronary artery disease and chest pain were examined, using DSE as usual, as well as TDI imaging of the mitral annulus at the septal, lateral, inferior, anterior, posterior regions and the proximal anteroseptal region from the apical views, before and immediately after DSE. In 24 subjects the study was normal, while wall motion abnormality was seen in 26, 9 of them only after DSE. Mitral annular systolic velocity at the 6 locations increased significantly after DSE both in normal subjects and in those with wall motion abnormality (WMA). After DSE mitral annular septal systolic velocity in normals, 19.2 ± 3.8 cm/sec, was higher than in those with WMA, 14.6 ± 2.5 cm/sec, P < 0.0003. Post-DSE mitral systolic velocity was senstive and accurate in predicting WMA.

**Conclusions:**

Systolic mitral TDI velocities increase after DSE, however to a lesser extent in those with wall motion abnormality, and can differentiate them from normal subjects.

## Introduction

Dobutamine stress echocardiography is an established method for the assessment of coronary artery disease with a sensitivity and specificity of 80% - 85% [1, 2], comparable to those of radio-isotopic myocardial perfusion studies. The major limitations of dobutamine stress echocardiography are lack of suitable acoustic window [3], suboptimal delineation of the endocardial border necessitating harmonic and contrast imaging [4-7], the subjective nature of analysis and the lack of uniform diagnostic criteria requiring high level of expertise in wall motion analysis [8-11].

The most important diagnostic element in dobutamine stress echocardiography is segmental systolic performance in the radial direction. However, abnormalities of longitudinal systolic performance may precede radial abnormalities and thus their evaluation may enhance the sensitivity of detection of contractile abnormalities [12, 13]. Tissue Doppler imaging has been shown to correlate with segmental myocardial wall motion [14-16].

Sampling at the mitral valve annulus, tissue Doppler imaging may reflect longitudinal systolic performance of the left ventricle.

The purpose of this study was to correlate immediate post dobutamine stress echocardiography regional mitral annular tissue Doppler imaging indices with regional left ventricular wall motion abnormalities during dobutamine stress echocardiography and their contribution to diagnsois.

## Methods

### Population

Fifty patients, 29 women, age 60.3 ± 13.4 years, were prospectively evaluated for the presence of coronary artery disease. All had transthoracic echocardiography before dobutamine stress echocardiography. Coronary angiography was performed according to clinical indications taking into consideration the dobutamine stress echocardiography (DSE) results.

### Dobutamine stress echocardiography

The protocol of dobutamine infusion consisted of 3 minute stages for each dose, staring with 5 µg/kg/min and increasing to 10, 20, 30 and 40 µg/kg/min. If end-points did not occur or 85% of the age adjusted heart rate was not achieved, 0.25 mg atropine was injected every 2 minutes up to 1 mg or until the target heart rate was achieved. Blood pressure and 12 lead electrocardiograms were recorded at rest and throughout the dobutamine stress echocardiography study. Horizontal or down-sloping > 1 mm ST-segment depression at 0.06 sec after the J point was considered as evidence for myocardial ischemia.

### Image acquisition

Images were obtained while the patients in the left lateral decubitus position. A standard commercial Hewlett Packard Sonos 5500 echocardiographic machine equipped with S4 transducer and second harmonic imaging as well as color-kinesis was used. Parasternal long axis and short axis as well as apical 4-chamber and 2-chamber views were recorded at rest, low dose dobutamine infusion, peak exercise and in the recovery period. Digital images were stored on magneto-optic discs for later off-line analysis. In addition super VHS videotape recordings were performed throughout the studies.

### Dobutamine stress echocardiographic analysis

Segmental left ventricular wall motion analysis was performed using 16-segment model [17]. Regional wall motion was estimated as normal, hypokinetic, akinetic or dyskinetic. Analysis of the gray-scale two-dimensional image as well as the color-kinesis overlay was combined in the assessment of regional left ventricular wall motion. New or worsening segmental wall motion was considered as ischemic response. Ischemic response (I) was identified when wall motion decreased by at least 1 grade in 2 adjacent segments or wall motion decreased by at least 2 grades in 1 segment, otherwise no ischemia, or normal response (N) was diagnosed.

### Tissue Doppler imaging

The apical 4 chamber, 2 chamber and 3 chamber views were used to assess the longitudinal velocities of the mitral annulus. The sample volume of the pulsed wave Doppler was located at the mitral annulus and recording was performed from the septal, lateral, anterior, inferior and posterior portions of the annulus. In addition Doppler sampling from the proximal anteroseptal segment was performed. Annular Doppler velocities were recorded on videotape for off-line analysis. Resting annular longitudinal velocities (S0), and immadiate post- dobutamine stress echocardiography peak systolic velocities (S1) were measured, and their difference (dS), dS/S0 and ratio (S1/S0) were calculated.

### Statistical analysis

Mean values and standard deviation of the 6 annular mitral longitudinal velocities, S0, S1, dS, dS/S0 and S1/S0 were calculated for all. Student t-test assuming unequal variances, was performed; P < 0.05 was considered significant.

## Results

All subjects underwent DSE studies safely and uneventfully. Heart rate increased from 62.4 ± 9.9 bpm to 123 ± 14.4 bpm. Systolic blood pressure increased from 138 ± 7 mmHg to 162 ± 9 mmHg.

### Left ventricular wall motion abnormalities

In 24 patients the dobutamine stress echocardiography results were considered normal (N), without baseline or dobutamine induced wall motion abnormalities. In 26 left ventricular wall motion abnormalities were observed (Pt), in 9 only after dobutamine infusion (Pt1), while resting wall motion abnormalities were observed in 17, with apical involvement in 5, antrior-septal in 2, inferior in 13, posterior in 4 and lateral in 1. In patients with resting wall motion abnormalities, in 2 (Pt2) wall motion improved after dobutamine stress echocardiography, in 3 (Pt3) wall motion did not change after dobutamine stress echocardiography, and in 12 (Pt4) worsened after dobutamine stress echocardiography (Table 1).

**Table 1:** Left Ventricular Wall Motion Score Index at Rest and After Stress Echocardiography in Subgroups of Wall Motion Abnormality

	WMSI-Rest	WMSI-Stress	HR Rest	HR Peak
Pt	1.166 ± 0.21	1.34 ± 0.18	62.4 ± 9.88	123.77 ± 14.38
Pt1	1 ± 0	1.33 ± 0.13	62.67 ± 10.79	130 ± 15.96
P value (Pt : Pt1)	**0.00053**	0.85634	0.950696	0.283587
Pt2	1.19 ± 0.09	1.06 ± 0.88	60 ± 7.07	118 ± 11.31
P value (Pt : Pt2)	0.800539	0.059406	0.7385	0.586514
Pt3	1.58 ± 0.32	1.58 ± 0.32	61.67 ± 15.57	109.33 ± 18.82
P value (Pt : Pt3)	0.154403	0.325027	0.90601	0.120262
Pt4	1.18 ± 0.12	1.34 ± 1.24	62.83 ± 9.33	123.67 ± 10.68
P value (Pt : Pt4)	0.761902	0.913018	0.90437	0.98257
Pt1 + Pt4	1.1 ± 0.13	1.34 ± 0.12	62.76 ± 9.7	126.4 ± 13.23
P value (Pt : Pt1 + Pt4)	0.43	0.113	0.45	0.26

Pt: All 26 subjects with WMA

Pt1: Subjects with WMA only after DSE

Pt2: Subjects with WMA at rest and improved after DSE

Pt3: Subjects with WMA at rest and did not changed after DSE

Pt4: Subjects with WMA at rest and worsened during DSE

### Mitral annular systolic velocities at rest

Generally, velocities were similar in normal subjects and in those with wall motion abnormality (WMA), except for slightly higher inferior and posterior mitral annular velocities in normal subjects (about 1 cm/sec higher) which, however, reached statistical significance (Table 2).

**Table 2:** Mitral Annular Systolic Velocities at Rest and After Dobutamine Stress Echocardiography in All Subjects

	Mean ± STD	septum	lat	inf	ant	post	antseptum	
Normals	Systolic velocity at rest (cm/sec)	11.1 ± 1.6	13 ± 2.3	12.2 ± 1.8	11.7 ± 2.5	12.4 ± 1.8	10.1 ± 1.3	
	Systolic velocity after stress (cm/sec)	19.2 ± 3.3	18.7 ± 3.6	17.97 ± 3.8	16.5 ± 4.3	17.95 ± 3	15.8 ± 3.5	
	N: P value Rest : Stress	4.15E-14	4.92E-08	1.97E-08	1.91E-05	6.06E-10	1.68E-09	
WMA(*)	Systolic velocity at rest (cm/sec)	10.7 ± 1.3	12 ± 1.6	11.2 ± 1.6	10.8 ± 1.4	11.1 ± 1.4	9.6 ± 1.3	
	Systolic velocity after stress (cm/sec)	14.6 ± 2.5	16.7 ± 3.4	15.9 ± 3.2	15.1 ± 4.1	16.4 ± 2.6	14 ± 3.2	
	WMA: P value Rest : Stress	3.83E-09	4.32E-08	1.6E-08	4.54E-06	4.21E-12	2.39E-08	
P value Normal : WMA(*)	Systolic velocity at rest (cm/sec)	0.385106	0.072574	0.034469	0.128962	0.009679	0.203429	
	Systolic velocity after DSE (cm/sec)	2.25E-6	0.050644	0.038527	0.23492	0.05088	0.065086	

(*) WMA = Wall motion abnormality

### Systolic mitral annular velocities after dobutamine stress echocardiography

After dobutamine stress echocardiography, mitral annular systolic velocities increased significantly in normal subjects and those with WMA, in all 6 regions of Doppler sampling (Table 2). However, only septal peak annular velocities after dobutamine stress echocardiography (Septal-S1) were significantly higher in normal subjects (Table 2).

### Calculated parameters of systolic mitral velocities

Post-dobutamine stress echocardiography and baseline velocity difference (dS = S1 - S0), and ratio (S1/S0) as well as dS/S1 were significantly higher in normal subjects compared to those with wall motion abnormalities (Table 3).

**Table 3:** Mitral Annular Systolic Velocity Calculated Parameters at Rest and After Dobutamine Stress Echocardiography in All Subjects

		septum	lat	int	ant	post	antseptum
dS	Normals	8.1 ± 3.14	5.7 ± 3.7	5.8 ± 3.33	4.8 ± 2.9	5.6 ± 2.5	5.74 ± 3.6
	WMA	3.87 ± 2.25	4.77 ± 3.23	4.69 ± 2.66	4.27 ± 3.5	5.26 ± 2.3	4.39 ± 2.9
	P value (N : WMA)	**2.79E-06**	0.347103	0.219606	0.562961	0.624443	0.156402
dS/S0	Normals	0.75 ± 0.34	0.47 ± 0.33	0.48 ± 0.29	0.41 ± 0.24	0.47 ± 0.22	0.59 ± 0.37
	WMA	0.37 ± 0.23	0.4 ± 0.29	0.43 ± 0.24	0.39 ± 0.31	0.48 ± 0.23	0.46 ± 0.299
	P value (N : WMA)	**4.13E-05**	0.523187	0.456129	0.833189	0.778375	0.194333
S1/S0	Normals	1.75 ± 0.34	1.47 ± 0.33	1.48 ± 0.29	1.41 ± 0.24	1.47 ± 0.22	1.59 ± 0.37
	WMA	1.33 ± 0.35	1.41 ± 0.29	1.43 ± 0.24	1.39 ± 0.31	1.48 ± 0.23	1.46 ± 0.29
	P value (N : WMA)	**7.7E-05**	0.523187	0.456129	0.833189	0.778375	0.194333

S0: Systolic velocity at rest (cm/sec)

S1: Systolic velocity after DSE (cm/sec)

dS: S1 - S0

dS/S0: ratio

S1/S0: ratio

### Relation of mitral annular systolic velocities to different groups of timing of wall motion abnormality

As shown in Table 4 systolic mitral annular velocities were normal at rest in all subgroups with wall motion abnormality. After DSE mitral annular systolic velocities were lower than normal in all subjects with WMA, except those without change in wall motion (Pt3). Combining the 21 subjects with ischemic response (Pt1 and Pt4) revealed that sytolic septal annular velocities were lower than normal after DSE.

**Table 4:** Mitral Annular Systolic Velocities at Rest and After Dobutamine Stress Echocardiography in Different Subgroups of Change in Wall Motion Abnormality

		septum	lat	inf	ant	post	antseptum
Pt1 (subjects with WMA only after DSE)	Systolic velocity at rest	10.87 ± 1.34	11.76 ± 2.06	16.9 ± 3.69	10.79 ± 1.48	10.4 ± 1.44	9.7 ± 1.55
	P value (N : Pt1)	0.69675	0.15398	0.10018	0.19628	**0.02306**	0.54178
	Systolic velocity after stress	14.3 ± 2.65	16.43 ± 2.34	16.14 ± 6.25	16.22 ± 4.83	16.3 ± 3.03	14.49 ± 3.64
	P value (N : Pt1)	**0.00039**	**0.04428**	0.23442	0.86926	0.18459	0.363378
Pt2 (subjects with WMA at rest and improved after DSE)	Systolic velocity at rest	11.15 ± 1.63	12.65 ± 0.21	11.6 ± 1.69	12.35 ± 2.33	12.2 ± 0.14	11 ± 0
	P value (N : Pt2)	0.96563	0.48167	0.71058	0.78023	0.69484	**0.00167**
	Systolic velocity after stress	17.05 ± 0.21	18.2 ± 5.37	17.5 ± 2.12	16.95 ± 1.06	15.2 ± 0.71	15.6 ± 0.57
	P value (N : Pt2)	0.00564	0.91552	0.80624	0.72899	**0.01318**	0.80086
Pt3 (subjects with WMA at rest and did not chang after DSE)	Systolic velocity at rest	10.6 ± 1.64	11 ± 0.4	10.9 ± 1.65	11.5 ± 2.04	11.37 ± 2.33	9.93 ± 1.29
	P value (N : Pt3)	**0.675163**	0.001023	0.288473	0.869686	0.55201	0.868895
	Systolic velocity after stress	14.9 ± 3.72	16.5 ± 5.77	15.33 ± 4.73	15.67 ± 5.90	15.7 ± 3.89	12.57 ± 3.07
	P value (N : Pt3)	0.200295	0.58228	0.45039	0.829103	0.43644	0.188043
Pt4 (subjects with WMA at rest and worsened during DSE)	Systolic velocity at rest	10.583 ± 1.24	12.23 ± 1.49	11.13 ± 1.92	10.4 ± 0.88	11.03 ± 1.32	9.23 ± 1.02
	P value (N : Pt4)	0.3074	**0.23317**	0.11806	**0.03138**	**0.01883**	**0.039109**
	Systolic velocity after stress	14.31 ± 2.21	16.75 ± 3.66	15.51 ± 2.72	13.83 ± 3.098	16.74 ± 2.33	13.74 ± 3.26
	P value (N : Pt4)	**1.18E-05**	0.140344	**0.03286**	**0.038515**	0.19485	0.093108
Pt1 + Pt4 (all subjects with worse WMA after DSE)	Systolic velocity at rest	10.7 ± 1.26	12 ± 1.73	11.2 ± 1.67	10.6 ± 1.15	11 ± 1.34	9.43 ± 1.26
	P value (N : Pt4 + Pt1)	0.187	0.057	**0.02**	0.026	0.0025	0.048
	Systolic velocity after stress	14.3 ± 2.34	16.6 ± 3.1	15.8 ± 3.1	14.85 ± 4	16.55 ± 2.59	14.06 ± 3.35
	P value (N : Pt4 + Pt1)	**5.83E-07**	**0.02**	**0.019**	0.09	0.05	**0.04**

### Relation of mitral annular systolic velocities to different groups of location of wall motion abnormality

Systolic mitral septal annular velocities at rest in subejcts with wall motion abnormality at different locations were similar to normal. After DSE mitral annular septal systolic velocities were less than normal in all the groups with wall motion abnormalities at all locations (Table 5).

**Table 5:** Mitral Annular Systolic Velocities at Rest and After Dobutamine Stress Echocardiography According to Location of Wall Motion Abnormality Compared to Normal

		septum	lat	inf	ant	post	antseptum
WMA lat	Systolic velocity at rest	10.75 ± 1.69	11.3 ± 2.61	10.7 ± 2.16	10.48 ± 0.98	10.9 ± 1.88	10.2 ± 2.17
	P value	0.7122	0.28568	0.25436	0.102278	0.22409	0.91791
	Systolic velocity after DSE	13.16 ± 3.18	13.5 ± 3.57	14.78 ± 4.1	12.28 ± 2.42	14.98 ± 3.26	12.1 ± 2.89
	P value	**0.025677**	0.05386	0.218365	**0.02454**	0.162935	0.06997
WMA inf	Systolic velocity at rest	10.52 ± 1.13	12.15 ± 1.51	11.03 ± 1.46	10.61 ± 1.18	10.92 ± 1.08	9.35 ± 0.95
	P value	0.173157	0.152316	**0.02069**	0.06015	**0.002898**	**0.038125**
	Systolic velocity after DSE	14.32 ± 2.28	16.58 ± 2.95	15.72 ± 3.11	14.94 ± 4.25	16.29 ± 2.47	14.16 ± 3.23
	P value	**1.51E-06**	**0.038304**	**0.03795**	0.23025	0.05339	0.11599
**WMA ant**	Systolic velocity at rest	10.3 ± 1.80	10.33 ± 2.14	10.27 ± 2.42	10.03 ± 0.51	10.47 ± 2.04	10.27 ± 2.65
	P value	0.546108	0.1366	0.30985	**0.009865**	0.266757	0.913125
	Systolic velocity after DSE	13.4 ± 1.29	15.75 ± 8.84	14.13 ± 4.77	12.83 ± 2.63	14.73 ± 3.95	11.3 ± 2.95
	P value	0.13139	0.17168	0.311784	0.1252	0.306382	0.09256
WMA post	Systolic velocity at rest	10.48 ± 1.02	11.78 ± 1.91	11.09 ± 0.59	10.39 ± 1.08	10.76 ± 0.95	9.09 ± 0.94
	P value	0.227	0.139556	**0.010516**	**0.039644**	**0.00307**	**0.02527**
	Systolic velocity after DSE	14.59 ± 2.16	16.49 ± 2.48	16.68 ± 3.49	14.31 ± 2.89	16.41 ± 2.79	13.63 ± 2.47
	P value	**0.00012**	0.05679	0.368676	0.09697	0.18818	0.05932
WMA apex	Systolic velocity at rest	10.72 ± 1.59	12.13 ± 1.34	11.32 ± 1.62	11.17 ± 1.38	11.73 ± 1.79	10.1 ± 1.16
	P value	0.62475	0.24804	0.26814	0.47069	0.47243	0.96421
	Systolic velocity after DSE	15.78 ± 2.55	16.98 ± 4.05	17.38 ± 3.75	16.37 ± 3.83	16.07 ± 3.17	13.95 ± 2.89
	P value	**0.022146**	0.36996	0.7406	0.92985	0.22995	0.211088

P value: versus Normal

### Relation to wall motion score index

**Figure 1:**
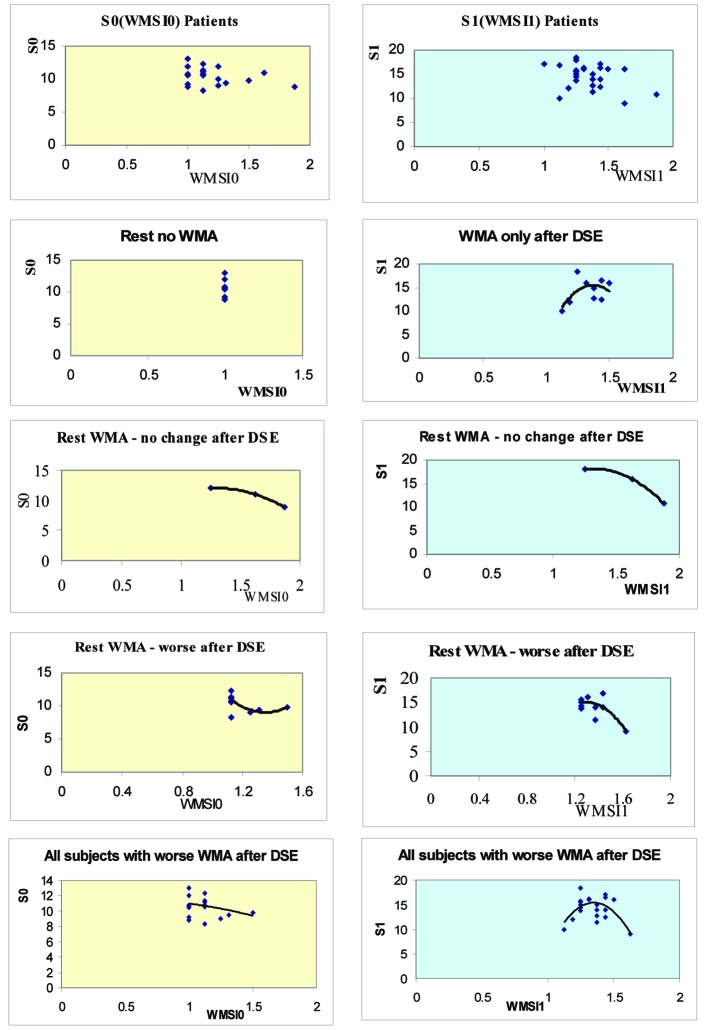
Mitral annular systolic velocity relationship with left ventricular wall motion score index at rest (left) and after dobutamine stress (right), in different groups of patients as depicted in each figure. S0: Systolic velocity at rest (cm/sec), S1: Systolic velocity after DSE (cm/sec), WMSI0: Wall motion score index at rest, WMSI1: Wall motion score index after DSE.

In subjects with wall motion abnormalities, an inverse relationship between baseline septal systolic velocity S0 and post-dobutamine stress echocardiography velocity S1 on one hand and wall motion score index on the other was observed (Fig. 1). Septal velocity began to decrease when wall motion score index exceeded 1.25. The same inverse relationship was obsereved in ptaients who developed wall motion abnormalities only after dobutamine stress echocardiography (Pt1), in those with baseline wall motion abnormalities which did not change during dobutamine stress echocardiography (Pt3), and in those with resting wall motion abnormalities which worsened during dobutamine stress echocardiography (Pt 4). In all these patient subgroups, systolic septal annular velocities decreased when wall motion score index exceeded 1.25. Subjects with ischemic response (Pt1 and Pt4 combined together) showed the same finding. Moreover, the increase in septal velocity after dobutamine stress echocardiography (dS), as well as (dS/S0) decreased when the difference in wall motion score index (WMSI) after dobutamine stress echocardiography was larger (Fig. 1).

### Diagnostic value of systolic septal annular velocities

**Figure 2:**
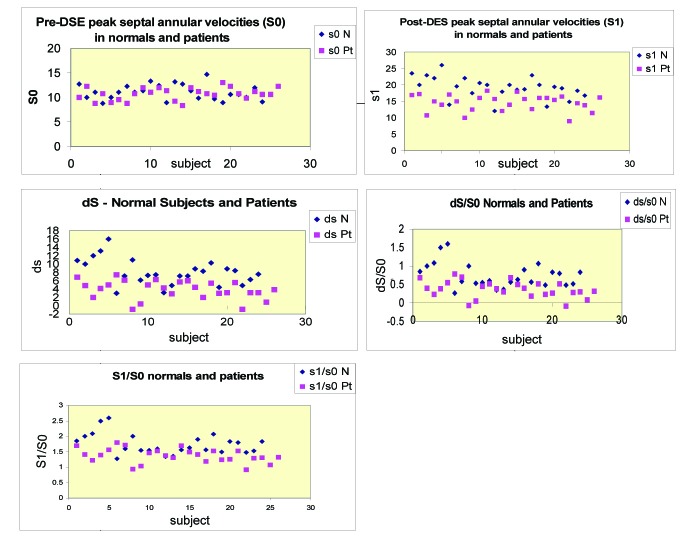
Scattergrams of mitral annular systolic velocities in normal subjects (rhomboids) and in patients with left ventricular wall motion abnormality (squares) at rest and after stress.

Baseline septal velocities (S0) did not differentiate between normal subjects and those with wall motion abnormalities (Fig. 2). However, post-dobutamine stress echocardiography septal velocities (S1), as well as velocity differnece (dS), (dS/S0) and (S1/S0) effectively differntiated normal subjects from those with wall motion abnormalities, with narrow band of overlap, as seen in Figure 2. Cutoff values with high sensitivty, specificity and diagnostic accuracy in detecting patients with wall motion abnormalities were achieved with S1 < 17 cm/sec, S1/S0 < 1.5, dS < 6 cm/sec and dS/S0 < 0.55 (Table 6).

**Table 6:** Diagnostic Value of Mitral Annular Systolic Velocity Parameters for the Detection of Wall Motion Abnormality

	Sensitivity (%)	Specificity (%)	Diagnostic Accuracy (%)
S1 < 17	92	80	88
S1/S0 < 1.5	85	88	86
dS < 6	88	79	84
dS/S0 < 0.55	85	79	82

S0: *Syst*olic velocity at rest (cm/sec)

S1: Systolic velocity after DSE (cm/sec)

dS: S1 - S0

dS/S0: ratio

S1/S0: ra*tio*

### Relationship of region of wall motion abnormality and systolic septal velocity

Septal velocities after dobutamine stress echocardiography lower than normal were observed when the wall motion abnormalities involved the left ventricular apex, anteroseptal, lateral and inferior segments (Table 5).

Only in those with anterior wall motion abnormalities no differnece was obeserved most probably due to small number of subjects.

### Relation to coronary angiography

Coronary angiography was not performed in subjects without wall motion abnormality and without ischemic response. In 12 subjects with wall motion abnormality coronary angiography was performed and stenotic lesion locations correlated with sites of regional wall motion abnormality.

## Discussion

The aim of the present was to examine the utility of longitudinal left ventricular systolic function from mitral annular tissue Doppler velocities in evaluation of dobutamine stress echocardiographic studies. It was found that mitral annular systolic velocities increased after dobutamine stress echocardiography in those with or without left ventricular wall motion abnormalities. In subjects with left ventricular wall motion abnormalities, the increase in septal annular systolic velocities after dobutamine stress was blunted. Systolic mitral annular velocity less than 17 cm/sec was sensitive, specific and accurate in predicting presence of wall motion abnormalities. Other calculated indices relating post-dobutamine stress echocardiography systolic mitral annular velocity to baseline values exhibited similar behavior.

Myocardial ischemia results in alteration of diastolic and systolic left ventricular function. The sequence of regional changes in myocardial function induced by acute ischemia was defined by experimental sonomicrometric techniques [18, 19]. Accordingly, detection of the presence of myocardial ischemia includes delay in onset of myocardial thickening, decrease in the rate and degree of thickening, late systolic thinning followed by delay in peak thickening and post-systolic thickening. The findings of this study relate to differences in the changes in the rate of longitudinal left ventricular systolic function represented by peak mitral annular systolic velocity.

Despite the widespread use of dobutamine stress echocardiography, evaluation of wall motion is still mostly visual and subjective resulting in inter-observer variability [8-11]. Optimal technique of evaluation should not only quantify the parameters of regional myocardial ischemia, velocities, amplitude and timing of motion but also should be simple in order to gain widespread application.

Several imaging methods have been introduced to make analysis of stress echocardiography more quantitative and less subjective [20-23]. However some of these may lack simplicity and cannot be applied by many commercially available echocardigraphic machines. In this study, simple numerical parameter-mitral annular systolic velocity, could predict presence of wall motion abnormalities making evaluation of stress echocardiographic studies more objective, although predicting localization of wall motion abnormalities was not effective. Mitral annular systolic velocity emerged as a parameter of global ischemia not related to the region of ischemia.

Tissue Doppler imaging methods have been applied for the detection of myocardial ischemia [24-24]. In normal subjects the segmental response to increase in dobuatmine infusion is a gradual and continuous increase in myocardial velocities, strain rate and strain [15, 27-29]. Consistent with our study it was reported that abnormal increase in segmental velocity during stress indicates ischemia [30, 31].

Mitral annular systolic velocity at the site insertion of the anterior mitral leaflet with the inter-ventricular septum was the most valuable region of evaluation of tissue Doppler velocities resulting in significant differences in velocities after stress between subjects with and those without left ventricular wall motion abnormality. When left ventricular wall motion score index was equal or greater than 1.25, septal mitral annular Doppler velocity was reduced as a marker of global left ventricular systolic dysfunction.

### Strengths and limitations

The strength of this study is that a simple evaluation of mitral annular tissue Doppler velocity after dobutamine stress echocardiography and related calculated were accurate in predicting left ventricular wall motion abnormality. However these parameters were not related to the site of wall motion abnormality, and instead they were related to global systolic function and dysfunction like increase in left ventricular wall motion score index more than 1.25. Another limitation was that coronary angiography was not performed in all subjects, therefore correlation with this hard gold standard was not possible.

### Conclusion

Systolic mitral TDI velocities increase after DSE, however to a lesser extent in those with wall motion abnormality, and can differentiate them from normal subjects. Application of TDI mitral velocities may simplify and aid in interpreting dobutamine stress echo studies.
